# Comparison of the Efficacy of Vaginal Hyaluronic Acid to Estrogen for the Treatment of Vaginal Atrophy in Postmenopausal Women: A Systematic Review

**DOI:** 10.7759/cureus.44191

**Published:** 2023-08-27

**Authors:** Nada Saleh Albalawi, Maram Ati Almohammadi, Ahmad Raja Albalawi

**Affiliations:** 1 Geriatrics Department, King Fahad Specialist Hospital, Ministry of Health, Tabuk, SAU; 2 Training Department, Academic Affairs and Training Administration, Ministry of Health, Tabuk, SAU; 3 Diabetic and Endocrine Department, King Fahad Specialist Hospital, Ministry of Health, Tabuk, SAU

**Keywords:** postmenopausal, estrogen, hyaluronic acid, dyspareunia, atrophic vaginitis

## Abstract

Topical estrogen is effective for treating postmenopausal vaginal atrophy. However, there is a potential risk of estrogen-related adverse effects. There is a need for finding effective non-hormonal treatment for vaginal atrophy. The topical application of moisturising agents, such as hyaluronic acid (HA), represents a promising non-hormonal treatment for the relief of vaginal atrophy. This study aimed to summarize the evidence regarding the efficacy of topical HA compared to topical estrogen in postmenopausal women with vaginal atrophy. The literature search covered English-published studies from database inception till February 2023. The search included the electronic databases of MEDLINE/PubMed, Cochrane Library, Web of Science, ProQuest, and Scopus, using the terms "Hyaluronic Acid" AND "Postmenopause" AND "Vagina” AND "Atrophy". Due to the diversity in reporting outcomes, meta-analysis was not feasible. A narrative synthesis with a systematic approach was conducted by vote counting of studies that included a direct comparison between topical HA and topical estrogen. Six studies were included. Intra-group comparisons showed that both interventions were significantly effective in alleviating the symptoms of vaginal atrophy and dyspareunia as well as improving vaginal pH and cell maturation index. However, inter-group comparisons in most studies showed that estrogen was superior to HA in relieving vaginal symptoms and improving vaginal pH, dyspareunia, and the cell maturation index. There is no evidence to show the superiority of HA to estrogen in the treatment of postmenopausal vaginal atrophy. However, the therapeutic efficacy of HA seems to be comparable to estrogen and considering its safety, HA can be used as an alternative to estrogen in patients who do not want to use estrogen. The available studies have several limitations, and the reporting of outcomes was considerably heterogeneous.

## Introduction and background

Menopause is defined as the permanent cessation of menses following amenorrhea that lasts 12 consecutive months without the use of hormonal contraception [1]. Menopause usually occurs naturally, but it may occur due to the depletion of ovarian follicles as a result of bilateral oophorectomy, radiation to the ovaries, or chemotherapy [2].

Decreased estrogen level after menopause leads to loss of cell proliferation in the vaginal epithelium, causing connective tissue damage with subsequent infiltration of leukocytes and macrophages [3]. The infiltration of inflammatory cells could reduce the vaginal vascular supply and lubrication of the vaginal epithelium [4]. In addition, epithelial vaginal cells die and exfoliate resulting in the loss of glycogen and decreased lactobacillus population accompanied by increased vaginal pH [5]. All these effects result in thinning of the vaginal epithelium with increased susceptibility to trauma [6]. These changes cause a condition known as vulvovaginal atrophy, which is experienced by nearly half the postmenopausal women [4].

Vulvovaginal atrophy constitutes an integral part of the genitourinary syndrome of menopause [6,7]. Most women with vulvovaginal atrophy complain of vaginal dryness, dyspareunia, irritation, burning, itching, discharge, urinary discomfort, and bleeding during sexual intercourse [7,8]. Thus, the condition negatively impacts both sexual function [9] and the overall quality of life [10] of postmenopausal women. Physical examination and laboratory tests can elicit signs that characterize vulvovaginal atrophy such as the appearance of the vaginal mucosa, changes in vaginal pH and the maturation index of vaginal epithelial cells [4,10].

The North American Menopause Society stated that the treatment of vulvovaginal atrophy aims primarily to relieve subjective symptoms. First-line agents include non-hormonal, long-acting vaginal moisturizers [11]. In women with poor response to hormone-free agents, the use of low-dose vaginal estrogen preparations, vaginal dehydroepiandrosterone (DHEA), systemic estrogen therapy, and ospemifene could effectively improve moderate to severe cases [11].

Although the topical application of estrogen to the vagina proved its efficacy in reducing the atrophic vaginal changes and alleviating the symptoms [12], there is a potential - yet unproven - increased risk of hormone-dependent cancers, heart disease, and stroke [13]. This explains the wide use of non-hormonal lubricants and moisturizers by postmenopausal women [14,15].

There is a plethora of non-hormonal vaginal products that can improve the moisturization of the vagina and alleviate the vulvovaginal symptoms [16]. Some vaginal products contain hyaluronic acid (HA), which is a high-molecular-weight polysaccharide. The moisturizing effect of HA is attributed to its binding with large amounts of water in the extracellular matrix [17]. The binding of HA gel to the vaginal wall can last up to three days and then is lost through the peeling of the epithelial cells [18,19]. Studies have demonstrated the effectiveness of HA in the treatment of vaginal atrophy without the concomitant use of hormonal products [20-22].

The present systematic review was conducted to summarize the evidence regarding the efficacy of topical HA compared to topical estrogen in postmenopausal women with vaginal atrophy.

## Review

Materials and methods

Methodology

This systematic review was conducted and reported according to the principles of the Cochrane Handbook for Systematic Reviews of Interventions, version 6 and the Preferred Reporting Items for Systematic Reviews and Meta-Analyses (PRISMA) guideline [23].

Research Question

Is topical HA as effective as topical estrogen preparations for the treatment of vaginal atrophy in postmenopausal women?

Research Aim and Objectives

This systematic review aimed to compare the efficacy of topical HA and topical estrogen in postmenopausal women with vaginal atrophy. The studied objectives we to compare the improvement in the symptoms of vaginal dryness between HA and topical estrogen and to compare the improvement in vaginal pH and maturation index of vaginal cells.

Eligibility Criteria for the Studies

This systematic review included comparative observational (cohort or case-control) studies and clinical trials. The literature search was limited to studies published in English from the database inception to February 2023. Studies were included if the studied subjects were postmenopausal women diagnosed with vaginal atrophy. Studies were eligible if they included a direct comparison between topical HA and topical estrogen regarding the alleviation of the symptoms of vaginal atrophy and dyspareunia as well as the changes in vaginal pH and vaginal cell maturation. Animal studies and studies that did not include a direct comparison between topical HA and topical estrogens were excluded. We also excluded conference abstracts, duplicate records, case reports, reviews, commentaries, editorials, and clinical guidelines.

Search Strategy

The search included the electronic databases of MEDLINE/PubMed, Cochrane Library, Web of Science, ProQuest, and Scopus. The search covered all publications from database inception till February 2023. The search was conducted in February 2023. The used search terms included "Hyaluronic Acid" AND "Postmenopause" AND "Vagina” AND "Atrophy" and no filters were used. The first author screened the reference lists of retrieved articles by electronic search in order to find other potentially relevant studies.

Selection of Studies

Literature search and screening of the titles and abstracts were conducted by the first author. The first author retrieved the full text of potentially relevant articles and assessed the studies’ eligibility for inclusion in this systematic review. The second author checked the search process, the screening of the records, and the selection of the studies. Any disagreements were settled by consulting the third author.

Data Extraction

The first author extracted data from the eligible studies using a standardized data sheet. The extracted data included (a) the characteristics of the study (the country, study design, time span, sample size, the inclusion and exclusion criteria, and the duration of follow-up); (b) patients’ characteristics (age at the time of study as well as the age at menopause or the duration of amenorrhea); (c) the intervention and control treatments: form, concentration, and regimen; and (d) the outcomes: the symptoms of vaginal dryness (itching, burning, and dyspareunia), vaginal pH, and maturation index of the vaginal cells after treatment. The second author revised the extracted data to ensure consistency and clarity. Any disagreements between the first and second authors were settled by consulting the third author. The third author revised the extracted data and checked the methods used for data synthesis.

Measured Outcomes

The primary outcome was an improvement in the symptoms of vaginal atrophy (itching, burning, and dyspareunia). The secondary outcomes were the change in vaginal pH and the maturation index of vaginal epithelial cells.

Assessment of the Risk of Bias in the Included Studies

We used the National Institute for Health and Care Excellence (NICE) checklists for randomized controlled clinical trials [24], cohort studies [25], and case-control studies [26] to assess the risk of bias (ROB) in eligible studies. The first author assessed the ROB and the second author revised the checklists.

Data Synthesis

There were wide variations among the included studies in reporting the studied outcomes, with some studies reporting vaginal symptoms as a composite score while other studies reported each symptom separately. Also, vaginal pH and the maturation index were sometimes reported as numerical variables and at other times as categorical variables. Consequently, pooling the data through the meta-analysis methods was not feasible. We performed a narrative synthesis with a systematic approach for the studied outcomes. A narrative synthesis table was created to report - for each outcome - the number of studies showing statistically significant effects and the direction of effect. The data were combined using the narrative synthesis methods by vote counting the direction of effect (i.e., by counting the number of studies favouring either treatment).

Results

Results of Literature Search and Study Selection

**Figure 1:**
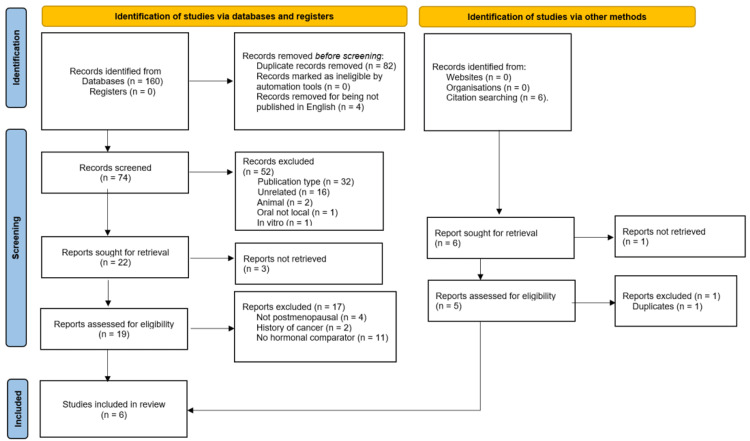
The PRISMA Flow Chart Diagram for the Results of the Literature Search and Study Selection PRISMA: Preferred Reporting Items for Systematic Reviews and Meta-Analyses

The conduction of the literature search yielded 160 records. Eighty-two records were duplicates and removed. Four records were published in languages other than English and were excluded. Then, we screened the titles and abstracts of the remaining 74 records, with the exclusion of 52 records due to the publication type (n = 32), non-relevance (n = 16), conduction on animals (n = 2) or in vitro (n = 1), and oral administration of HA (n = 1). The full texts of the remaining 22 records were sought, with the successful retrieval of 19 records while the full text of three records could not be obtained. The retrieved 19 records were assessed for eligibility to be included in this systematic review. Seventeen records were not eligible; 11 studies lacked a comparison group, or the comparator did not contain estradiol, four studies were conducted on premenopausal women, and two studies included patients with a history of cancer. Searching the reference lists of retrieved full texts yielded six potentially relevant records, out of which two were excluded (one was a duplicate and the other lacked an estradiol comparator). Finally, six studies were included in this systematic review (Figure 1) [21,27-31].

Basic Characteristics of the Included Studies

All studies were randomized controlled trials (RCT), except one study [27], which was a quasi-randomized clinical trial. The studies were conducted in Turkey [30], China [28], Iran [21], Brazil [29], and Italy [27,31]. HA was used in the form of tablets [30], suppositories [31], gel [27,28], and cream [21,29]. The used estrogens included estradiol [29,30], estriol [27,28], conjugated estrogens [21], and genistein which is a phytoestrogen [31]. The used concentrations of both agents varied widely across the studies, as well as the dose regimen. The duration of follow-up varied from three weeks up to 15 weeks or more (Tables 1, 2).

**Table 1:** Characteristics and Interventions of the Included Studies (N = 6) E: estrogen; HA: hyaluronic acid; NR: not recorded; RCT: a randomized controlled trial

Study	Study design	Country	Time span	Sample size Total (HA: E)	HA form and regimen	Estrogen form and regimen	Follow-up (weeks)
Ekin et al. 2010 [30]	RCT	Turkey	NR	42 (21 : 21)	HA sodium salt vaginal tablets (5 mg) once daily for 8 weeks	Estradiol vaginal tablets (Vagifem) 25 mcg; once daily for 14 days and then twice weekly	8
Le Donne et al. 2011 [31]	RCT	NR	NR	62 (31 : 31)	HA vaginal suppositories (5 mg) daily for 15 consecutive days/month for 3 months	Intravaginal suppositories 97 mcg of genistein daily for 15 consecutive days/month for 3 months	12
Chen et al. 2013 [28]	RCT	China	May 2009 to May 2010	133 (67 : 66)	HA Intravaginal gel (Hyalofemme) 5 g every 3 days for 3 weeks (10 doses)	Estriol in vaginal cream (Ovestin) 0.5 g every 3 days (for 10 doses)	4
Jokar et al. 2016 [21]	RCT	Iran	Sept 2013 to Mar 2014	56 (28 : 28)	HA sodium salt Vaginal cream (Shiraz Pharmacy College) 5 mg/d for 8 weeks	Conjugated estrogen vaginal cream (Aborayhan Pharmaceutical Company) 0.625 mg/d for 14 days then twice weekly for 6 weeks	8
Duque-Estrada et al. 2017 [29]	RCT	Brazil	Oct 2015 to Nov 2015	68 (35 : 33)	HA Vaginal cream (Lubrinat®) once daily twice a week for 3 consecutive weeks	Estradiol vaginal cream (Colpotrofine) once daily for 3 weeks	3
Alvisi et al. 2022 [27]	Quasi-randomized clinical trial	Italy	Jan 2019 to Jan 2020	50 (25 : 25)	HA vaginal gel 0.2% (HYALOGYN; Fidia Farmaceutici, Abano Terme, Italy) for 21 days then 3 times per week + non-ablative CO2 laser in 3 laser sessions performed 4 to 6 weeks apart	Estriol vaginal gel (GELISTROL) 50 mcg daily for 21 days then 3 times per week + non-ablative CO2 laser in 3 laser sessions performed 4 to 6 weeks apart	15 ± 4.2

**Table 2:** Eligibility Criteria ad Patients’ Characteristics in the Included Studies (N = 6) E: estrogen; HA: hyaluronic acid; NR: not recorded; numeric variables were reported as mean ± standard deviation

Study	Age	Duration of menopause (years)	Age at menopause (years)	Inclusion criteria	Exclusion criteria
Ekin et al. 2010 [30]	HA: 52.95 ± 4.80 E: 51.86 ± 4.35	HA: 4.67 ± 3.13 E: 5.29 ± 3.03	NR	Sexually active women aged ≥45 years or older; moderate to- severe vaginal dryness & soreness; serum E2 concentrations ≤ 20 pg/mL; superficial vaginal cells ≤ 5% or less; postmenopausal ≥ 12 months; endometrial thickness ≤ 5 mm on transvaginal US	History of breast cancer or hormone-dependent tumour; genital bleeding of unknown cause; acute thrombophlebitis or estrogen-related thromboembolic disorder; vaginal infection; allergy to the test drug; disease interfering with study compliance; Investigational drug within 30 days before screening, homoeopathic preparation within 7 days before study drug initiation, or exogenous corticosteroid/sex hormones within 8 weeks before study drug initiation
Le Donne et al. 2011 [31]	HA: 59.7 ± 3.4 E: 57.8 ± 4.7	NR	HA: 49.5 ± 0.7 E: 48.5 ± 4.9	Postmenopausal for ≥ 2 years; symptomatic and cyto-colposcopically evident vaginal atrophy	Atypical cervical vaginal smears; hormonal therapy or vaginal treatment in the previous 6 months
Chen et al. 2013 [28]	HA: 54.05 ± 4.27 E: 54.41 ± 4.60	HA: 4.44 ± 3.71 E: 5.58 ± 5.45	NR	Age <70 years old; naturally or surgically postmenopausal >6 months; symptoms of vaginal dryness; no contraindication to local estrogen	Unmarried, pregnant, & breast-feeding women; vaginal infections; breast cancer, uterine cancer, or estrogen-dependent tumours; genital bleeding of unknown cause; acute hepatopathy, embolic disorders, severe 1ry renal or bone marrow disease; recent malignant tumours; use of vaginal products within 1 week, estrogens within 1 month, and other investigational products within 2 weeks of the study start
Jokar et al. 2016 [21]	HA: 56.4 ± 5.47 E: 51.92 ± 4.31	NR	HA: 47.71 ± 5.26 E: 46.2 ± 4.16	Married & menopause women; moderate to severe vaginal dryness; endometrial thickening ≤5mm on vaginal US	Smokers; anticoagulant drugs; topical hormonal and non-hormonal drugs 1 month before study; vaginal infection; sensitivity to drug; history of hormone-related diseases (e.g., breast cancer, unknown cases of vaginal bleeding); severe thrombophlebitis or thromboembolism; chronic diseases (e.g., cardiac diseases, hypertension, diabetes)
Duque-Estrada et al. 2017 [29]	HA: 56.7 ± 5.7 E: 55.2 ± 5.5	NR	NR	Able to consent; postmenopausal women; Age between 40 and 70; vaginal dryness with intact skin and mucosa; Concordance to adhere to the procedures and follow-up	Allergy to the tested products; skin condition in the product application area; Skin diseases like psoriasis, vitiligo, and atopic dermatitis; Immunologic failure; Diabetes Mellitus Type 1; Diabetes-related complications (e.g., retinopathy, nephropathy, neuropathy) or dermatoses (e.g. plantar ulcer, lipoid necrobiosis, granuloma annulare, opportunistic infections); History of hypoglycemia, diabetic ketoacidosis and/or hyperosmolar coma; Current topical or systemic use of corticosteroids, immunosuppressant drugs, and antihistamine; Other disorders or medication that may interfere directly in the study or endanger the participant's health
Alvisi et al. 2022 [27]	HA: 57.2 ± 8.5 E: 57.2 ± 7.0	HA: 8.4 ± 6.9 E: 6.9 ± 5.5	HA: 48.9 ± 6.9 E: 50.5 ± 3.3	Vaginal Health Index score <15; amenorrhea for ≥12 months; recent negative cervical smear test and signed informed consent to treatment and to the anonymous use of data	Use of systemic or local hormonal therapy or ospemifene within 6 months before study; acute urinary tract infections; previous use of energy-based devices; contraindications to estrogen therapies; vaginal bleeding of unknown origin

Evidence Synthesis of the Included Studies

Ekin et al. [30] carried out an RCT on postmenopausal sexually active women with moderate to severe vaginal dryness and soreness. The study arms consisted of 21 patients in the HA group (5 mg HA sodium salt) and 21 in the estrogen group (25 ug estradiol). Both treatments were used for eight weeks. A 4-point scale composite score was used to assess the symptoms of vaginal atrophy. They categorized the degree of epithelial atrophy as none, mild, moderate, and severe. Comparison of before-after symptoms, vaginal atrophy, vaginal maturation values, and vaginal pH within each group showed a significant improvement in both groups (P < 0.001). The estrogen group showed a significantly more reduction in symptoms as well as a significant improvement in pH and vaginal maturation values compared to the HA group (P <0.05). They included that both treatments were effective in alleviating vaginal symptoms as well as reducing epithelial atrophy and increasing vaginal pH and maturation of the vaginal epithelium. However, estrogen was superior to HA.

Le Donne et al. [31] carried out an RCT on postmenopausal women with symptomatic and cyto-colposcopically evident vaginal atrophy. The study arms consisted of 31 patients in the HA group (5 mg HA vaginal suppositories) and 31 in the estrogen group (97 ug of genistein). Both treatments were applied daily for 15 consecutive days/month for three months. Both groups showed a significant improvement in genital symptoms, colposcopy scores, and maturation value (p<0.001). The improvement in genital score was significantly higher in genistein than in the HA group (p < 0.001), but not in the maturation value (p = 0.271). They concluded that both treatments improved genital symptoms and maturation value, but genistein was more effective in relieving the symptoms.

Chen and co-workers [28] carried out an RCT during the period from May 2009 to May 2010 naturally or surgically postmenopausal women under the age of 70 years. The study arms consisted of 67 in the HA group (5 g HA intravaginal gel) and 66 in the estrogen group (0.5 g estriol in vaginal cream). Both treatments were applied every three days for 10 doses. Both groups showed a significant improvement in the clinical symptoms of vaginal dryness. The improvement rates were 84.44% and 89.42% in the HA and estrogen groups, respectively, without statistically significant differences between the two groups. They concluded that HA is a valid alternative to estrogen-based treatments, with nearly equal efficacy.

Jokar et al. [21] conducted their RCT during the period from September 2013 to March 2014 on married menopausal women with moderate-to-severe vaginal dryness. Each group consisted of 28 patients. HA was administered in the form of 5 mg HA sodium salt vaginal cream and was applied daily for eight weeks. The control group received 0.625 mg conjugated estrogen vaginal cream once daily for two weeks then twice per week for six weeks. There was a significant improvement in vaginal pH, dyspareunia, and the composite score of the vaginal symptoms compared with the pre-treatment values within both groups (P<0.001). The HA group showed improvement in urinary incontinence as well (P<0.05). Between-group comparisons showed that HA was superior to estrogen in improving urinary incontinence, dryness, maturation index (P<0.05), and the composite score of vaginal symptoms (P<0.001).

Duque-Estrada et al. [29] carried out an RCT on postmenopausal women whose ages ranged between 40 and 70 and suffered vaginal dryness with intact skin and mucosa. The study was conducted from October 2015 to November 2015. Thirty-five patients applied HA vaginal cream once daily twice a week, while 33 patients used an estradiol vaginal cream once daily. Both treatments were applied for three consecutive weeks. They observed the lack of significant difference between the HA gel and the estrogen cream as regards the dryness of the vaginal mucosa (p = 0.786) as well as the self-reported moisturizing effect (p = 0.142), comfort sensation (p = 0.528), and the fragrance of the product (p = 0.088). They concluded that both preparations were comparable, and this supported the use of HA as a first-line treatment of the symptoms of vaginal dryness before prescribing estrogen-containing products.

Alvisi et al. [27] conducted a quasi-randomized clinical trial during the period from January 2019 to January 2020 on postmenopausal women with a Vaginal Health Index score <15. The patients were categorized into three groups (25 patients each). The first group underwent treatment with a non-ablative laser only in three sessions performed four to six weeks apart, while the second and third groups received in addition to the laser treatment either estrogen or a moisturizing cream containing HA. HA was administered as 0.2% vaginal gel for 21 days, then the gel was applied three times per week. Estrogen was administered in the form of a 50-mcg estriol vaginal gel daily for 21 days, it was applied three times per week. They found that one month after the last laser session, there was a significant within-group improvement in the mean vaginal health index and vulvar health index, as well as the VAS scores of dryness, burning and itching, with no significant inter-group differences. Similarly, sexual function improved significantly in all groups compared to baseline, without significant inter-group differences. However, laser+HA showed a significantly higher improvement in the lubrication domain of the Female Sexual Function Index.

Assessment of the Risk of Bias in the Included Studies

As regards the selection bias, the ROB was uncertain in three studies [29-31] due to a lack of description of the process of randomization and allocation concealment. In addition, epithelial atrophy was more severe in the HA group than in the estrogen group at the beginning of the study by Ekin et al. [30]. The study by Duque-Estrada et al. [29] did not compare the baseline characteristics of the patients, except for age, so we cannot ascertain whether the two groups were comparable before the interventions started. A high risk of selection bias was evident in the study by Alvisi et al. [27], as no proper randomization was carried out and the groups were not comparable at baseline (longer duration of amenorrhea in the laser group and higher percentage of sexually active women in the laser+estrogen group). As for the performance ROB, equal care apart from the intervention was provided for the two groups. None of the studies reported whether the patients or carers were blinded to the treatment, and three studies stated clearly that they were open-label [21,28,29]. The overall risk of performance bias was unclear in one study [30], high in three studies [21,28,29], and low in two studies [27,31]. As for the attrition ROB, the overall risk was low in four studies [21,27,30,31], uncertain in one study [29] due to insufficient information about the numbers completing treatment and high in another study [28] for a considerable number not completing the assessment or treatment. Regarding the detection of ROB, all studies stated a length of follow-up that was below the 12-week duration recommended by the Food and Drug Administration (FDA) [32], except for two studies [27,31]. All studies used appropriate methods for assessing the outcomes. Blinding of the assessors was reported by three studies [21,30,31], while was uncertain in two studies [27,29] and not done in one study [28]. However, as the assessed outcomes by the investigators were objectively measured (e.g., vaginal pH and the maturation index), the ROB is not expected to be considerable with unblinding of the outcome assessors. The overall detection ROB was high in one study [28], uncertain in two studies [27,29], and low in the remaining three studies (Table 3) [21,30,31].

**Table 3:** The Risk of Bias Assessment for the Included Studies Based on the NICE Tools for Clinical Trials and Cohort Studies A1: An appropriate method of randomisation was used to allocate participants to treatment groups (which would have balanced any confounding factors equally across groups); A2: There was adequate concealment of allocation (such that investigators, clinicians and participants cannot influence enrolment or treatment allocation); A3: The groups were comparable at baseline, including all major confounding and prognostic factors; B1: The comparison groups received the same care apart from the intervention(s) studied; B2: Participants receiving care were kept "blind" to treatment allocation; B3: Individuals administering care were kept "blind" to treatment allocation; C1: All groups were followed up for an equal length of time (or analysis was adjusted to allow for differences in length of follow-up); C2a: How many participants did not complete treatment in each group?; C2b: The groups were comparable for treatment completion; C3a: For how many participants in each group were no outcome data available?; C3b: The groups were comparable with respect to the availability of outcome data; D1: The study had an appropriate length of follow-up; D2: The study used a precise definition of outcome; D3: A valid and reliable method was used to determine the outcome; D4: Investigators were kept "blind" to participants' exposure to the intervention; D5: Investigators were kept "blind" to other important confounding and prognostic factors.

	Ekin et al. 2010 [30]	Le Donne et al. 2011 [31]	Chen et al. 2013 [28]	Jokar et al. 2016 [21]	Duque-Estrada et al. 2017 [29]	Alvisi et al. 2022 [27]
A1	Uncertain	Uncertain	Yes	Yes	Uncertain	No
A2	Uncertain	Uncertain	Yes	Uncertain	Uncertain	No
A3	No	Yes	Yes	Yes	Uncertain	No
selection bias	Uncertain	Uncertain	Low	Low	Uncertain	High
B1	Yes	Yes	Yes	Yes	Yes	Yes
B2	Uncertain	Uncertain	No	No	No	Uncertain
B3	Uncertain	Yes	No	No	No	Yes
Performance bias	Uncertain	Low	High	High	High	Low
C1	Yes	Yes	Yes	Yes	No	Yes
C2a	None	One	5 & 6	None	One & one	None
C2b	Yes	Yes	Yes	Yes	Yes	Yes
C3a	None	None	Considerable	None	Uncertain	None
C3b	Yes	Yes	No	Yes	Uncertain	Yes
Attrition bias	Low	Low	High	Low	Uncertain	Low
D1	No	Yes	No	No	No	Yes
D2	Yes	Yes	Yes	Yes	Yes	Yes
D3	Yes	Yes	Yes	Yes	Yes	Yes
D4	Yes	Yes	No	Yes	Uncertain	Uncertain
D5	Yes	Yes	No	Yes	Uncertain	Uncertain
Detection bias	Low	Low	High	Low	Uncertain	Uncertain

Vaginal Atrophy Symptoms and Vaginal pH

The six studies reported a change in the symptoms of vaginal atrophy after receiving treatment. Different methods were used to describe vaginal symptoms in the studies, as most studies used a composite score [21,29-31], while one study reported a percentage improvement [28] and another study used the Visual Analog Scale (VAS) [27]. Four studies reported that both HA and estrogen significantly alleviated the symptoms as indicated by intra-group comparisons [21,27,30,31], while the remaining two studies did not conduct intra-group comparisons [28,29]. Vote counting showed that two studies [21,27] out of the six reported that HA was superior to estrogen preparations in relieving the symptoms of vaginal dryness and the result was significant in the study by Jokar et al. [21]. Out of the four studies favouring estrogen, two showed a statistically significant difference [30,31], while in the other two studies, the difference did not reach statistical significance [28,29]. Three studies reported a change in vaginal pH after receiving treatment [21,28,30]. Measurement of pH was performed by inserting an indicator band in the vagina. Vaginal pH was reported by two studies as a categorical variable [21,30] and the third study [28] expressed vaginal pH as a numerical variable. A fourth study [27] assessed vaginal pH within the Vaginal Health Index and did not report pH individually. Two studies reported a significant improvement in vaginal pH in intra-group comparisons [21,30], while the study by Chen et al. [28] did not conduct intra-group comparisons. Vote counting showed that one study [21] out of the three reported a more beneficial effect of HA, but the result was not statistically significant. The other two studies reported that estrogen was significantly superior to HA in improving vaginal pH (Table 4) [28,30].

**Table 4:** Summary of the Improvement in Vaginal Symptoms and pH in the Included Studies (N = 6) E: estradiol; HA: hyaluronic acid; NR: not recorded

Study	Symptoms of vaginal atrophy				Vaginal pH			
	Outcome assessed	Reported data	Vote counting	Outcome assessed		Reported data	Vote counting	
Ekin et al. 2010 [30]	Composite score of vaginal symptoms Mean (SD)	3.86 (1.39) vs. 2.67 (1.53) P=0.012	Favours estrogen	pH categories (<5, 5-5.49, & 5.-6.49)		HA: 0%, 71.4%, 28.6% E: 14.2%, 85.7%, 0% P=0.003	Favours estrogen	
Le Donne et al. 2011 [31]	Genital score (median)	2 vs. 1, p=0.001	Favours estrogen	NR		NR	-	
Chen et al. 2013 [28]	the percentage improvement Mean (SD)	Itching: 63.66 (38.25) vs. 67.29 (37.79), p=0.6613 Burning: 85.83 (24.63) vs. 87.87 (36.66), p=0.1072	Favours estrogen	pH measurements Mean		HA: 5.30 E: 4.87 P<0.05	Favours estrogen	
Jokar et al. 2016 [21]	Composite score of vaginal symptoms Mean (SD)	2.60±1.39 vs. 4.10±1.66, p<0.001	Favours HA	pH categories (<5, 5-5.49, 5.-6.49, & >6.49)		HA: 60.7%, 25.1%, 7.1%, 7.1% E: 42.9%, 21.4%, 21.4%, 14.3% P=0.463	Favours HA	
Duque-Estrada et al. 2017 [29]	Sum of a score on dryness Mean (SD)	97 (1) vs. 100, p=0.786	Favours estrogen	NR		NR	-	
Alvisi et al. 2022 [27]	Visual Analog Scale change Mean (95% CI)	Dryness: -2.6 (-3.7, -1.6) vs. -2.2 (-3.3, -1.1) Burning: -2.1 (-3.2, -1.1) vs. -1.7 (-2.8, -0.5) Itching: --1.4 (-2.4, -0.5) vs. -1.0 (-2.0, 0.0) p>0.05	Favours HA	NR		Measured within VHI which did not sig differ	-	
Synthesis			2/6 (33.3%)				1/3 (33.3%)	

Dyspareunia and Vaginal Cell Maturation

Three studies reported a change in vaginal pH after receiving treatment [27,28,30]. Dyspareunia was reported by one study as a categorical variable [30], while the other two studies [27,28] expressed the outcome as a numerical variable. Two other studies assessed dyspareunia as a component of a composite score that included also other symptoms of vaginal atrophy [21,31], but they did not report the outcome of dyspareunia separately. Two studies reported significant alleviation of dyspareunia in intra-group comparisons [27,30], while the study by Chen et al. [28] did not perform intra-group comparisons. Vote counting showed that one study [27] out of the three reported a more beneficial effect of HA, but the result was not statistically significant. The other two studies reported that estrogen was insignificantly superior to HA in improving vaginal pH [28,30]. Three studies reported on the change in the maturation of vaginal cells after receiving treatment [21,30,31]. The vaginal cell maturation index was assessed by cytological examination. The maturation index was reported by two studies as a categorical variable [21,30], while the third study expressed the outcome as a numerical variable [31]. The three studies reported a significant improvement in intra-group comparisons [21,30,31]. Vote counting showed that the three studies reported a more beneficial effect of estrogen compared to HA, reaching statistical significance in two studies (Table 5) [21,30].

**Table 5:** Summary of Dyspareunia and Cell Maturation in the Included Studies (N = 6) E: estradiol; HA: hyaluronic acid; NR: not recorded

Study	Dyspareunia				Vaginal cell maturation			
	Outcome assessed	Reported data	Vote counting	Outcome assessed		Reported data	Vote counting	
Ekin et al. 2010 [30]	Categories of severity (None, Mild, Moderate, & severe)	HA: 14.2%, 76.1%, 9.5%, 0% E: 42.8%, 57.3%, 0%, 0% P=0.05	Favours estrogen	Vaginal maturation value Mean (SD)		HA: 44.40 (9.32) E: 71.19 (12.96) P=0.001	Favours estrogen	
Le Donne et al. 2011 [31]	NR	NR	-	Maturation value Median		HA: 1.5 E: 2 P=0.271	Favours estrogen	
Chen et al. 2013 [28]	The percentage improvement Mean (SD)	HA: 56.96 (41.47) E: 62.33 (43.80) (p=0.2918)	Favours estrogen	NR		NR	-	
Jokar et al. 2016 [21]	NR	NR	-	Categories of severity (None, Mild, Moderate, & severe)		HA: 0%, 3.6%, 89.3%, 7.1% E: 0%, 0%, 89.3%, 10.7% P=0.018	Favours estrogen	
Duque-Estrada et al. 2017 [29]	NR	NR	-	NR		NR	-	
Alvisi et al. 2022 [27]	Change in Female Sexual Function Index (pain) Mean (95% CI)	HA: 1.3 (0.6, 2.0) E: 1.1 (0.5, 1.7) p>0.05	Favours HA	NR		NR	-	
Synthesis			1/3 (33.3%)				3/3 (100%)	

Discussion

Summary of the Main Findings

The present systematic review was conducted to summarize the evidence regarding the efficacy of topical HA compared to topical estrogen in postmenopausal women with vaginal atrophy. The search of the electronic databases and reference lists resulted in the inclusion of six eligible studies in this systematic review [21,27-31].

Several studies established the efficacy of topical estrogens in alleviating the symptoms and cellular changes of vaginal atrophy [12]. However, the potential (though unproven) risk of experiencing estrogen-related adverse effects with topical use warranted the search for other non-hormonal preparations [13]. Several non-hormonal vaginal products have been assessed and demonstrated their efficacy in improving symptoms of vaginal atrophy [16]. Among these alternatives, HA is a promising agent that demonstrated its efficacy and safety in several studies. However, most of these studies had a before-after design without a control group [33-36].

In a normal vagina, glycogen breakdown produces glucose, which is converted into lactic acid, resulting in a vaginal pH ranging between 3.5 and 4.5. In postmenopausal women who do not receive hormonal replacement therapy, glycogen content gradually decreases. Moreover, there is reduced exfoliation of the vaginal epithelium. These factors will result in increased vaginal pH and a decrease in the normal lactobacilli flora [37]. Therefore, the outcomes compared between topical estrogen and HA included the symptoms of vaginal dryness (itching, burning, and dyspareunia), vaginal pH, and maturation index (decrease of parabasal vaginal cells and increase in superficial vaginal cells) of the vaginal cells after treatment. The choice of the studied outcomes was in accordance with the FDA criteria which stated that studies should include within the primary efficacy endpoint the vaginal pH, vaginal maturation index and the most irritating moderate to severe symptoms [32].

The synthesis of this review indicated that both HA and estrogen were effective in relieving the symptoms of vaginal atrophy and dyspareunia as well as improving the vaginal pH and cell maturation index. However, estrogen was superior to HA in most studies. One study only reported that HA achieved significantly better outcomes than estrogen [21], while another study reported a lack of significant differences between the two groups, with insignificantly higher rates of improvement in the estrogen group [28].

Overall Completeness, Applicability, and Quality of the Evidence

This systematic review summarized the current evidence on the efficacy of topical HA compared to topical estrogen as a treatment for postmenopausal vaginal atrophy. The results show that HA is effective in alleviating the symptoms and improving the vaginal pH and maturation index to reach nearly pre-menopausal levels. However, the results suggest that the efficacy of HA is inferior to estrogen. Meanwhile, other factors - besides the degree of efficacy - should be considered when recommending treatment for postmenopausal vaginal atrophy. As HA combines satisfactory efficacy with safety, it can be prescribed as a first-line treatment, particularly in women with mild symptoms. The use of estrogens can be kept for patients with moderate to severe symptoms or for those who do not improve on non-hormonal moisturizing preparations. This inference supports the recommendations of the position statement of the North American Menopause Society [9].

However, the results of the present systematic review are limited by the observed limitations of the included studies. There are concerns regarding the selection and performance of ROB. Although the studies mentioned that randomization was performed, no details were reported to support this statement except in two studies [21,28]. The baseline patients’ characteristics were either not reported [29] or showed significant differences between the two groups [27,30]. No attempts were made to adjust for the statistically significant differences between the two groups. The blinding of patients and carers was either not done [21,28,29] or uncertain [27,30,31]. Attrition ROB was also an issue, particularly in the study by Chen et al. [28] in which missed patients’ data at assessment reached up to one-third of the patients. Issues related to the detection of ROB were also observed, as the duration for application of treatment was far below the recommended 12 weeks duration [32] in most studies. The non-blinding of outcome assessors was either clearly stated [28] or suspected [27,29], but the introduced ROB may not be large, as the assessed outcomes of vaginal pH and cell maturation index were objectively measured.

Furthermore, there was underreporting of the important outcomes of changes in vaginal pH [27,29,31] and cell maturation [27-29] by some studies. A bothersome symptom such as dyspareunia was also underreported [21,29,31].

In addition, we were unable to conduct a meta-analysis as the reporting of outcomes was heterogeneous across the studies, which is a common problem in studies investigating genitourinary symptoms [38]. We performed instead narrative synthesis using the method of vote counting. However, if a large number of studies were available with standardized reporting of the outcomes, meta-analysis would have been feasible and would have given better insights into the efficacy of the studied interventions.

A previous systematic review [39] addressed the same question as the present review, but it included only four of the studies assessed in our review. The fifth study included in the systematic review by Dos Santos et al. [39] compared HA to placebo, not to estrogen, so this study [20] was excluded from our review. In addition, the review by Dos Santos et al. [39] described the studies’ findings only, without conducting any of the narrative synthesis methods such as vote counting and the direction of effect. The previous and present reviews assessed the same outcomes and the conclusions of the two reviews are nearly similar and they support the use of HA as therapy for patients who object to using hormonal therapy.

## Conclusions

The topical application of HA was both effective and safe. Our results support the recommendations of the position statement of the North American Menopause Society that HA can be used as a first-line treatment, particularly in mild vaginal atrophy. Estrogens can be used with moderate to severe symptoms or in non-responders as estrogen is expected to be more effective than HA in those patients. The restricted use of estrogens is due to the potential risk of estrogen-dependent cancers, heart disease, and stroke. Also, the use of topical estrogen in the case of women who experienced estrogen-dependent cancer may not be advisable.

The limitations of the available studies warrant the conduction of future RCTs with adequate sample size, ample duration of follow-up and adherence to the criteria of high-quality RCTs, including proper conduction and reporting of randomization, allocation concealment, double-blinding, and complete reporting of the outcomes. The studies should endeavour to assess the FDA-recommended outcomes. In addition, future studies should explore whether certain patient-related factors (e.g., age, duration of amenorrhea, comorbidities) can impact the outcomes and perform subgroup analysis if such factors were identified.
